# Activity‐Directed Synthesis: A Flexible Approach for Lead Generation

**DOI:** 10.1002/cmdc.202000524

**Published:** 2020-09-04

**Authors:** George Karageorgis, Samuel Liver, Adam Nelson

**Affiliations:** ^1^ School of Chemistry and Astbury Centre for Structural Molecular Biology University of Leeds Leeds LS2 9JT UK; ^2^ Rosalind Franklin Institute Harwell Campus Didcot OX11 0FA UK

**Keywords:** lead generation, molecular diversity, reaction toolkit

## Abstract

Activity‐directed synthesis (ADS) is a structure‐blind, functional‐driven molecular discovery approach. In this Concept, four case studies highlight the general applicability of ADS and showcase its flexibility to support different medicinal chemistry strategies. ADS deliberately harnesses reactions with multiple possible outcomes, and allows many chemotypes to be evaluated in parallel. Resources are focused on bioactive molecules, which emerge in tandem with associated synthetic routes. Some of the future challenges for ADS are highlighted, including the realisation of an autonomous molecular discovery platform. The prospects for ADS to become a mainstream lead generation approach are discussed.

## Graphical abstract

**Parallel discovery** of diverse bioactive small molecules for a range of targets: Activity‐directed synthesis has enabled a range of medicinal chemistry strategies to be realised, including scaffold constraint; fragment‐based discovery; expansion of a series of ligands; and scaffold hopping. Future scientific challenges, and the prospects of activity‐directed synthesis becoming a mainstream lead generation approach, are discussed.

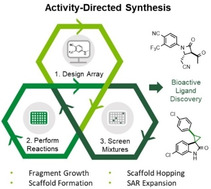

## Introduction

The discovery of bioactive small molecules is an enduring challenge in both medicinal chemistry and chemical biology. Lead generation is generally driven by iterative cycles in which series of molecules are designed, synthesised, purified and tested.^1^ Established lead generation approaches tend be both time‐ and resource‐intensive, in part because a similar investment is made in all molecules, irrespective of their ultimate biological activity. Although automation is widely harnessed within the individual stages of discovery workflows,^2^ it is rare for adjacent stages to be integrated, and for all activities (particularly purification) to be performed in parallel and with matched throughput. Moreover, a limited reaction toolkit dominates molecular discovery,^3^ which has led to an uneven exploration of chemical space and has tended to focus attention on molecules with suboptimal properties.^4^ Molecular design hypotheses are typically investigated one‐by‐one, focusing on individual series of closely related molecules that may be prepared using a specific reaction drawn from the standard toolkit. How, then, might diverse chemical space be explored more efficiently to drive the discovery of both drugs and chemical probes?

**Figure 1:**
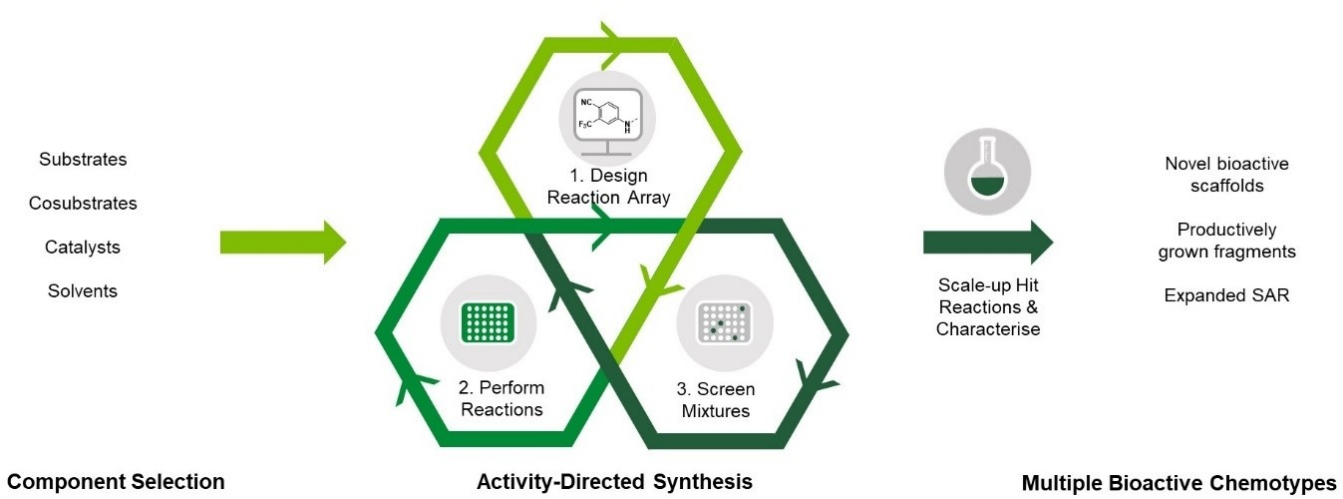
Overview of activity‐directed synthesis. Designed reaction arrays harness combinations of alternative components (such substrates, co‐substrates, catalysts and solvents). The crude reaction products are directly screened for biological function, and the identified hit reactions inform the design of subsequent reaction arrays. Finally, the most promising hit reactions are scaled up, and the products are purified, structurally elucidated and characterised.

Activity‐directed synthesis (ADS) is a structure‐blind, function‐driven approach in which active compounds emerge in parallel with an associated synthetic route (Figure 1). The approach borrows concepts from the evolution of biosynthetic pathways to natural products, a process that is driven by functional benefit to the host organism.^5^ A conceptually related approach termed “synthetic fermentation” also identifies compounds based on their biological activity, and enables the discovery of bioactive β‐peptides.^6^, ^7^ In ADS, reactions that have the potential to yield multiple possible products are deliberately exploited. Arrays of reactions are performed on a microscale (typically ∼10 μmol), and the resulting crude product mixtures are directly screened for biological function. A crucial step is to show that none of the individual components display activity in the screening assay. The reaction arrays may be assembled from stock solutions of components (e. g., substrates, co‐substrates, catalysts, solvents), typically using multi‐channel pipettes, via processes that are amenable to automation. After minimal work‐up, such as scavenging to remove metals, the crude product mixtures are diluted and screened for biological function. Here, as multiple products may be formed, screening is performed at a specific total concentration of the products formed from the limiting substrate. Hit reactions that yield bioactive products are thereby identified and can inform the design of subsequent reaction arrays. In subsequent rounds of ADS, screening may be performed at lower total product concentration in order to drive the emergence of reactions that yield more active product mixtures. Finally, the hit reactions are scaled up, and the responsible bioactive products are purified, structurally elucidated and characterised.

To allow exploration of diverse chemical space by ADS, it is important to harness reactions that have multiple (and diverse) possible outcomes. Here, the selection of underpinning chemistry contrasts starkly with that for conventional discovery workflows in which reactions that yield specific, designed molecules are preferred. Metal‐carbenoid chemistry has many of attributes that are required to underpin ADS.^8^ First, the diazo substrates can undergo many different types of reaction (e. g., O−H, N−H and C−H insertion; cyclopropanation; ylid formation) in either an inter‐ or an intramolecular sense. Second, the distribution of alternative products can often be tuned through variation of the metal catalyst. Such chemistry might therefore enable diverse chemical space to be explored, and the synthesis of bioactive chemotypes to be optimised.

In this Concept, we describe the reported applications of ADS that have led to the discovery of bioactive small molecules. We note the range of reaction classes and assay formats that have been used to underpin ADS. In each case, it is explained how ADS was exploited to realise alternative medicinal chemistry strategies. We discuss the value of the approach to allow diverse chemical space to be explored to yield distinctive starting points for molecular discovery. Finally, we comment on the future challenges that would need to be addressed in order for ADS to become a mainstream lead generation approach.

## Discovery of Androgen Receptor Modulators Ligands Based on Novel Scaffolds

**Figure 2:**
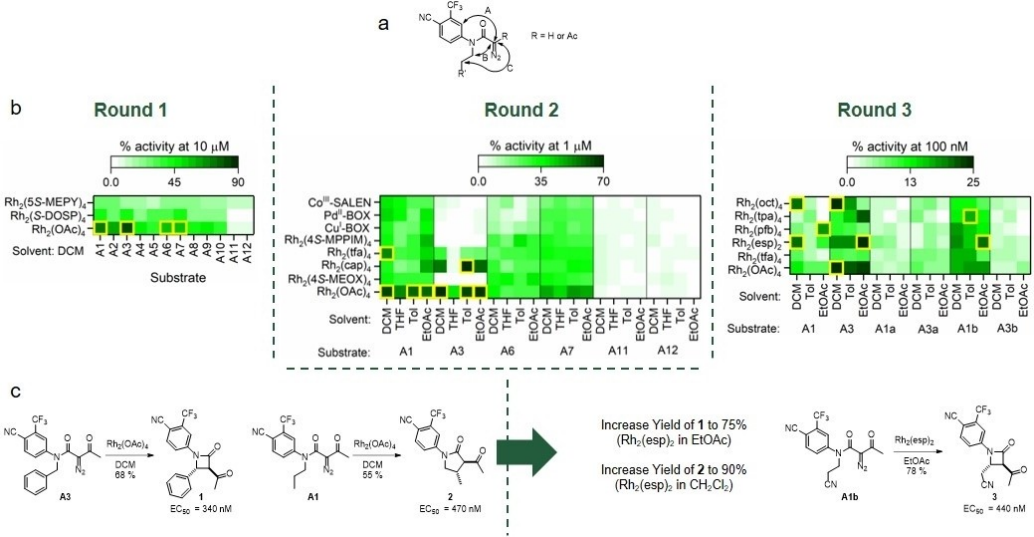
Activity‐directed discovery of AR modulators based on scaffolds with no previously annotated activity against AR. a) Potential cyclisation pathways of diazo substrates **A1**–**A12**. b) Activities of crude product mixtures formed in reaction arrays in rounds 1 (left), 2 (middle) and 3 (right), relative to 5 μM testosterone; hit reactions are highlighted (yellow boxes). c) Reactions discovered that yield AR modulators.

ADS was initially exploited in the discovery of androgen receptor (AR) modulators based on scaffolds with no previously annotated AR activity (Figure 2).^9^ AR is a nuclear receptor which acts as a transcription regulator, crucial for the expression and development of male sexual characteristics; mutations of the AR have been associated with prostate cancer and androgen sensitivity syndrome.^10^

In round 1, twelve α‐diazo amides (**A1**–**A12**) were harnessed that each incorporated a fragment (4‐cyano‐3‐trifluoromethyl‐phenyl) found in known AR ligands as well as a variable R’ group. It was envisaged that several different cyclisation pathways might be possible to yield products based on alternative scaffolds (Figure 2a). In the reaction array, all combinations of the twelve substrates (**A1**–**A12**) and three dirhodium catalysts (1 mol%) were investigated in a single solvent (CH_2_Cl_2_; Figure 2b, left). The crude products were scavenged to remove metals, evaporated, and screened for agonism of the AR using a FRET‐based assay (total product concentration: 10 μM in 1 % DMSO in pH 7.5 buffer). Four substrates (**A1**, **A3**, **A6**, and **A7**), in combination with Rh_2_(OAc)_4_, resulted in product mixtures with significant activity, and these combinations informed the design of a second reaction array.

In round 2, six diazo substrates were exploited: the four substrates identified in round 1, and two additional substrates (**A11** and **A12**) that had not yielded bioactive products. The six substrates were each treated with a metal catalyst (8 alternatives) in a specific solvent (4 alternatives). The crude product mixtures were scavenged and evaporated as before, and screened at tenfold lower concentration (total product concentration: 1 μM; Figure 2b, middle). It was observed that the most active product mixtures stemmed from two of the substrates (**A1** and **A3**) in combination with dirhodium carboxylate catalysts.

In round 3, the reaction array harnessed, in addition to **A1** and **A3**, four additional structurally related substrates (**A1a**, **b** and **A3a**, **b**). The substrates were each treated with a dirhodium carboxylate catalyst (6 alternatives) in a specific solvent (3 alternatives). After screening at tenfold lower concentration (total product concentration: 0.1 μM; Figure 2b, right), the most promising reactions were scaled up, and the products purified and structurally elucidated (Figure 2c). All three products were found to be sub‐micromolar modulators of AR: the agonists **1** and **3** and the partial agonist **2**. Crucially, none of the ligands were based on scaffolds with any previously annotated activity against AR.

## Fragment‐Based Discovery of Androgen Receptor Agonists

**Figure 3:**
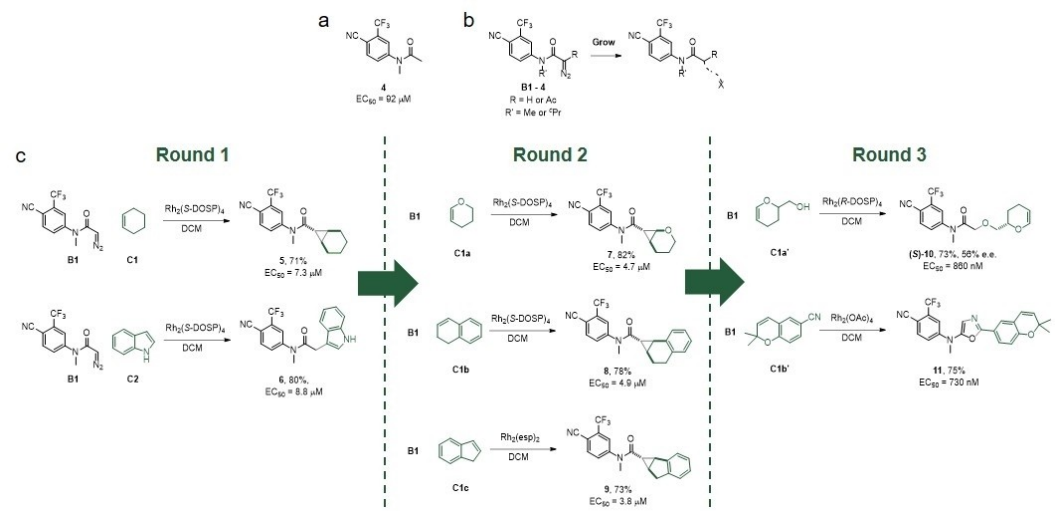
Activity‐directed discovery of AR modulators by fragment growth. a) Structure of a fragment that binds to AR. b) Potential intermolecular reactions of the diazo substrates **B1**–**4**. c) Evolution of the structure of AR modulators through three rounds of ADS.

ADS was exploited to identify productive strategies for the growth of **4**, a fragment that modulates AR (EC_50_=92 μM; Figure 3a and b).^11^ Accordingly, four diazo substrates (**B1**–**B4**) were designed based on this fragment, with R’ groups selected to minimise the possibility of intramolecular reaction.

In round 1, 192 of the 480 possible combinations of the 4 α‐diazo substrates (**B1**–**4**), 9 co‐substrates (**C1**–**9**; plus no co‐substrate), 6 dirhodium catalysts and two solvents were explored. After screening the product mixtures at a total product concentration of 10 μM, hit reactions were found that involved the diazo substrate **B1** and either cyclohexene (**C1**) or indole (**C2**; Figure 3c, left).

In subsequent rounds, the co‐substrates used were inspired by the productive co‐substrates from the previous round, and screening was performed at increasingly low total product concentration (Figure 3c, middle and right). In round 2, reactions of the diazo substrate **B1** with dihydropyran (**C1a**), dihydronaphthalene (**C1b**) or indene (**C1c**) were found to be productive; whilst, in round 3, reactions of **B1** with the substituted dihydropyran **C1a’** or the chromene **C1b’** were identified.

The hit reactions from all three rounds were scaled up, and the products purified, structurally elucidated and characterised. It would found that the fragment **4** could be productively grown by cyclopropanation (→**5**, round 1; →**7**, **8** or **9**, round 2), C−H insertion into indole (→**6**, round 1), O−H insertion (→**10**, round 3) or reaction with a nitrile (→**11**, round 3). ADS had thus enabled fragment growth to afford four distinct chemotypes in parallel, and the yields of bioactive products were always good. The activity of the fragment **4** had been improved ∼125‐fold in the case of the most active product **11** (EC_50_=730 nM).

## Expansion of a Series of Antibacterials

**Figure 4:**
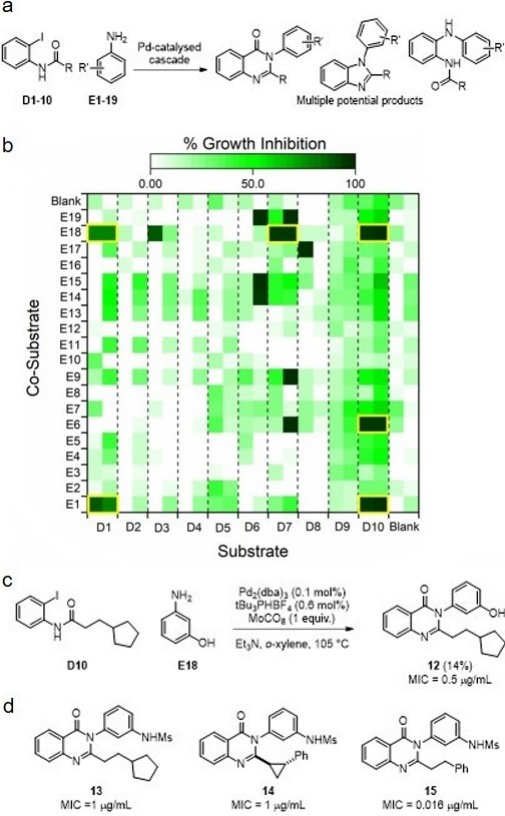
Activity‐directed expansion of a series of antibacterial quinazolinones. a) Potential Pd‐catalysed reaction pathways. b) Growth inhibition of crude product mixtures formed in the reaction array; hit reactions are highlighted (yellow boxes). c) Reaction that yielded the antibacterial quinazolinone **12**. d) Some other antibacterial quinazolinones that were identified following scale up.

ADS enabled expansion^12^ of a series^13^ of antibacterial quinazolinones. Pd‐catalysed cascade chemistry was chosen to drive ADS due to its potential to yield products based on many possible scaffolds (including quinazolinones; Figure 4a). Here, the alternative products stem from the possibility of cross‐coupling with or without carbonylation, and with or without subsequent cyclisation.

A reaction array of 220 reactions was executed based on all combinations of ten substrates (**D1**–**D10**; plus no substrate) and nineteen co‐substrates (**E1**–**E19**; plus no co‐substrate; see Figure 4c for conditions). The components were chosen to offer the possibility that multiple products might be formed, and to explore a diverse range of substitution patterns. After 48 h, the reactions mixtures were filtered through silica, evaporated, and screened against *S. aureus* ATCC29213 at a total product concentration of 50 μM (Figure 4b).

The products of six reactions displayed antibacterial activity against both replicate cultures: substrate **D1** with co‐substrates **E1** or **E18**; substrate **D7** with co‐substrate **E18**; and substrate **D10** with co‐substrates **E1**, **E6** or **E18**. The combination of **D1** and **E1** had previously been validated during the establishment of the ADS protocols, and therefore the remaining five reactions were scaled up and the products isolated, structurally elucidated and characterised. In each case, quinazolinones products were identified (e. g., **12**–**15**) which had minimum inhibitory concentrations (MIC) ranging from 0.016–1 μg/mL (Figure 4c and d). The study demonstrated that ADS could enable efficient expansion of the SAR of a series of antibacterials and enabled key features for antibacterial activity to be identified. At the 2‐position of the quinazolinone, a two‐atom linker to a hydrophobic substituent was essential and conformational restriction could be tolerated. At the 3‐position of the quinazolinone, a phenyl ring bearing a *meta* hydrogen donor substituent was required. The study additionally demonstrated the feasibility of ADS with a phenotypic assay and alternative underpinning chemistry.

## Discovery of Novel Inhibitors of the p53/hDM2 Protein–Protein Interaction by Scaffold Hopping

ADS facilitated the discovery of new scaffolds which enable the hot spot residues of a protein–protein interaction (PPI) to be targeted.^14^ It was shown that metal carbenoid chemistry could link fragments containing substituents that mimic hot spot residues of the p53 peptide to yield novel p53/*h*DM2 inhibitors.

An initial array of 154 reactions was performed using seven diazo substrates (**F1**–**F7**), ten co‐substrates (**G1**–**G10**; plus no co‐substrate) and two dirhodium catalysts. The diazo substrates and co‐substrates were designed to include substituents, such as phenyl, chlorophenyl and branched/cyclic/fluorinated alkyl groups, that may mimic hot spot residues of p53 peptide. Many of these groups are found in known p53/*h*DM2 inhibitors.

**Figure 5:**
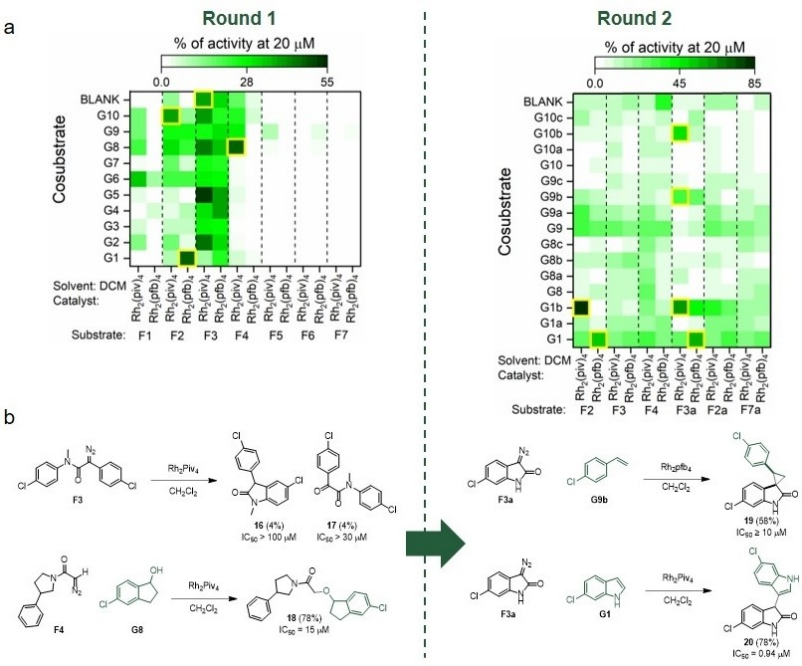
Activity‐directed discovery of p53/*h*DM2 PPI inhibitors. a) Activities of crude product mixtures formed in reaction arrays in rounds 1 (left) and 2 (right); hit reactions are highlighted (yellow boxes). b) Reactions discovered to yield p53/*h*DM2 PPI inhibitors.

After 24 h, the reactions were scavenged, evaporated, dissolved in DMSO and screened in duplicate at a total product concentration of 20 μM using an established fluorescence anisotropy (FA) assay (Figure 5a, left). Significant bioactivity was identified in the products of reactions with the diazo substrates **F2**, **F3** and **F4**. In the case of **F3**, activity was observed for reactions with or without a co‐substrate, suggesting that **F3** alone might yield bioactive product(s); the Rh_2_piv_4_‐catalysed reaction of **F3** was shown to yield both **16** and **17** (Figure 5b, left). LC/MS of three hit reactions involving **F2** and **F4** confirmed the formation of intermolecular reaction products in each case.

The intermolecular hit reactions from round 1 informed the design of a second array of 196 reactions. The array exploited six diazo substrates (including the new substrates **F3a**, **F2a** and **F7a**), 16 co‐substrates (including 11 new co‐substrates) and the same two dirhodium catalysts. After screening, six additional hit reactions were identified (Figure 5a, right).

Reactions with significant bioactivity from both rounds were scaled up 50‐fold, and the products purified and structurally elucidated. Products were identified from a range of reaction types (Figure 5b): intramolecular insertion into an aryl C−H bond (→**16**); insertion into an O−H bond (→**18**); cyclopropanation (→**19**); and insertion into an indoyl C−H bond (e. g., →**20**). The activity of the purified products was evaluated in the fluorescence anisotropy assay and were further validated as *h*DM2 binders by ^1^H,^15^N HSQC NMR spectroscopy.

The use of metal carbenoid chemistry to link substrates containing substituents with the potential to mimic p53 hot spot residues enabled four distinct and novel series of p53/*h*dm2 inhibitors to be discovered in parallel. It was concluded that **17**, **18**, **19** and **20** all contain pairs of substituents that target two hot spots. These ligands are highly dissimilar to each other, and to all ligands in ChEMBL with annotated activity against *h*DM2. Here, ADS enabled experimental scaffold‐hopping, resulting in the discovery of ligands in which common fragments were displayed in the context of alternative scaffolds. PPIs generally do not have defined small‐molecule binding sites, yet ADS was shown to identify distinctive starting points for the discovery of PPI inhibitors.

## Discussion

The above case studies showcase ADS as an efficient approach for the discovery of novel bioactive small molecules. ADS requires a robust high‐throughput assay, and both target‐oriented and phenotypic assays have been shown to be suitable (Table 1). Although over 200 reactions were conducted in each case, only a few reactions (typically <10) needed to be scaled up and the products purified and structurally elucidated. ADS thus focuses resources on reactions that yield bioactive products.

**Table 1:** Summary of activity‐directed synthesis case studies.

	Biological target (assay type)	Underpinning chemistry	Medicinal chemistry strategy	Number of rounds and reactions	Discoveries
**1** (Figure 2, 9)	Androgen receptor (FRET)	Metal carbenoid	Scaffold discovery	3 rounds (36, 192 and 108 reactions)	Agonists and partial agonists with sub‐micromolar activity Scaffolds with no previously annotated AR activity
**2** (Figure 3, 11)	Androgen receptor (FRET)	Metal carbenoid	Fragment growth	3 rounds (192, 86 and 48 reactions)	Four distinct chemotypes Elaborated fragments with low micromolar (or better) activity
**3** (Figure 4, 12)	*S. aureus* (cell‐based bacterial)	Pd‐catalysed carbonylation	SAR expansion	1 round (220 reactions)	Expanded series of antibacterial quinazolinones
**4** (Figure 5, 14)	p53/*h*DM2 PPI (fluorescence anisotropy)	Metal carbenoid	Scaffold hopping	2 rounds (154 and 192 reactions)	Experimental scaffold hopping to yield low micromolar PPI inhibitors Ligands for a binding site not evolved to bind small molecules

Retrospective analyses have provided insights into the mechanism of evolution of activity‐directed syntheses. The yield of the most active component may be optimised in an ADS workflow: for example, the yield of the AR partial agonist **3** increased significantly when a new catalyst was introduced.^9^ The focus on reactions which result in optimised yields of bioactive products (such as the elaborated AR agonists^11^ in Figure 3) can greatly facilitate subsequent purification. However, optimisation of the structure of a bioactive product is also possible: this can simply expand a series of ligands (e. g., AR agonists^11^ **7**–**9**, Figure 3 and antibacterial quinazolinones^12^ Figure 4), or it can lead to entirely new chemotypes (such as the AR agonists^11^ **10**–**11** and the PPI inhibitors^14^ **19**–**20**).

The use of a promiscuous, yet tuneable, reaction toolkit allows multiple diverse, yet synthetically accessible, chemotypes to be explored in parallel. This contrasts with overwhelming medicinal chemistry practice in which *perceived* synthetic accessibility tends to influence which molecules are prioritised for synthesis. In several cases, retrospective analysis showed that other chemotypes had been explored in ADS reaction arrays: for example, intramolecular insertion into an aryl C−H bond in substrates similar to **A3** gave oxindoles rather than lactams as products.^9^ These alternative chemotypes were not taken forward because other reactions had resulted in more active product mixtures.

ADS has enabled a range of medicinal chemistry strategies to be realised. It has enabled the discovery of ligands based on more constrained scaffolds (such as the AR modulators **1**–**3** 9); productive fragment growth (e. g., to give the AR agonists compounds **5**–**11** 11); expansion of compound series (e. g., the antibacterials **12**–**15** 12); and scaffold hopping (e. g., to give the p53/*h*DM2 inhibitors **17**–**20** 14). In each case, ADS enabled the parallel identification of distinctive starting points for bioactive molecular discovery.

## Future Prospects

ADS has the potential to become a mainstream lead discovery approach. It is highly general, requiring only access to a suitably robust high‐throughput assay. To date, substrate design has been informed by known ligands, for example by deconstruction of known inhibitors or harnessing the structures of fragment hits. However, exploiting larger, target‐agnostic reaction arrays, it may also be possible to discover wholly novel chemotypes. To manage the size of initial reaction arrays based on each reaction, whilst enabling diverse chemical space to be explored, we envisage that selected combinations of fragment‐sized substrates (perhaps chosen from <100 diverse alternatives) might be explored.

It may also be possible to harness late‐stage functionalisations to enable activity‐directed tuning of the potency, selectivity and/or molecular properties of ligands; recent advances in photoredox catalysis^15^ and C−H functionalisation^16^ chemistry may prove crucial for realising this goal. Additionally, we note the potential of other ligand‐directed synthetic approaches (in addition to those outlined in this Concept article) to explore in parallel small molecules based on multiple scaffolds.^17^

Finally, we believe that ADS has the potential to enable the autonomous discovery of bioactive small molecules. All stages of the workflow are performed in parallel and are amenable to integration and automation. Although human‐driven to date, it may be possible to develop algorithms to design reaction arrays that enable diverse, yet relevant, chemical space to be explored autonomously.

## Conflict of interest

The authors declare no conflict of interest.
